# Immune and Inflammatory Responses of the Intestinal Mucosa following Extended Liver Radiofrequency Ablation

**DOI:** 10.1155/2017/3450635

**Published:** 2017-10-25

**Authors:** Petros Ypsilantis, Maria Lambropoulou, Antonios Evagellou, Nikolaos Papadopoulos, Constantinos Simopoulos

**Affiliations:** ^1^Laboratory of Experimental Surgery and Surgical Research, School of Medicine, Democritus University of Thrace, Alexandroupolis, Greece; ^2^Laboratory of Histology and Embryology, School of Medicine, Democritus University of Thrace, Alexandroupolis, Greece

## Abstract

**Background and Aim:**

Extended liver radiofrequency ablation (RFA) has been shown to disrupt gut barrier integrity with subsequent bacterial translocation. The aim of the present project was to study the immune and inflammatory responses of the intestinal mucosa after extended RFA of the liver.

**Methods:**

Twelve Wistar rats were either subjected to RFA of the left lateral hepatic lobe (approximately 30% of the liver mass) after midline laparotomy (group RFA, *n* = 6) or sham operation (group Sham, *n* = 6). Forty-eight hours later, ileal tissue specimens were excised for immunohistochemical assessment of CD68^+^ macrophages, CD4^+^ T-lymphocytes, CD8^+^ T-lymphocytes, mucosal addressin cell adhesion molecule-1 (MAdCAM-1), tumor necrosis factor-*α* (TNF*α*), interleukin-6 (IL-6), and nuclear factor-*κ*B (NF*κ*B) expression.

**Results:**

Immune response biomarkers were upregulated in the RFA group. Expression of CD4^+^ and CD8^+^ T-lymphocytes was moderate, while that of CD68^+^ macrophages and MAdCAM-1 was high. Inflammatory response biomarkers were also upregulated in the RFA group. TNF*α*, IL-6, and NF*κ*B expression was low, moderate, and high, respectively.

**Conclusions:**

Extended liver RFA evokes both immune and inflammatory responses of the gut mucosa.

## 1. Introduction

The intestinal mucosa has to serve two opposite functions: the selective absorption of nutrients and the prevention of spread of intestinal microorganisms, luminal antigens, and proinflammatory factors to other organs and tissues. Contact of the intestinal mucosa with commensal bacteria stimulates a complex and highly specialized innate and adaptive immune system that creates a state of “physiological inflammation” and constitutes the so called “immune gut barrier” [1].

Disruption of the gut barrier, encountered in several pathologic conditions, such as hemorrhagic shock, trauma, obstructive jaundice, acute pancreatitis, and burn injury [2–6], leads to migration of intraluminal microorganisms through the intestinal epithelium to extraintestinal tissues. This mechanism has been proposed to lead to systemic infection and multiple organ dysfunction syndrome (MODS) [7, 8]. Invasion of the disrupted epithelial barrier by commensal bacteria exacerbates local immune and inflammatory reactions in order to prevent bacterial spread.

Radiofrequency ablation (RFA) of the liver has been widely accepted as an alternative local treatment of unresectable primary or metastatic tumors [9]. Although the method is considered safe, rare septic complications (2.4–4.6%) may increase morbidity and mortality [10, 11]. The incidence of infectious side-effects has been related to the volume of the ablated liver mass [10, 12]. There is evidence from experimental studies that extended liver RFA results to disruption of the mechanical and biological components of the intestinal mucosa barrier with subsequent translocation of bacteria and endotoxins [13, 14]. So far, studies of the histologic profile of the intestinal epithelium have revealed inflammation of mild severity [13, 15].

The aim of the present project was to study the immune and subsequent inflammatory responses of the intestinal mucosa in a rat model of extended liver RFA.

## 2. Methods

### 2.1. Animals

Twelve Wistar rats, 4 months of age, weighing 300–350 gr, provided from our laboratory's rat colony, were used. They were housed in polycarbonate cages, 3 rats per cage, under controlled environmental conditions (20–22° room temperature, 12 hour light: 12 hour dark cycle) and were provided with commercially available rat chow and tap water ad libitum.

### 2.2. Experimental Design

The animals were randomly assigned to 2 groups of 6 animals each and were subjected to either RFA of the left liver lobe (approximately 30% of total liver mass) after midline laparotomy (group RFA) or sham operation (group Sham). Forty-eight hours postoperation, tissue samples were excised from the terminal ileum for immunohistochemical evaluation of the expression of CD68^+^ macrophages, CD4^+^ T-lymphocytes, CD8^+^ T-lymphocytes, the mucosal addressin cell adhesion molecule-1 (MAdCAM-1), interleukin-6 (IL-6), tumor necrosis factor-*α* (TNF*α*), and nuclear factor-*κ*B (NF*κ*B). Finally, the animals were euthanized by exsanguination. The experimental protocol was approved by the Animal Care and Use Committee of the local veterinary service since it complied with Directive 86/609/EEC which was the legislation in force at the time of experimentation.

### 2.3. Preparation of Animals—Operation

The animals were anesthetized by administration of the inhaled anesthetic sevoflurane (2% in oxygen) via a face mask. A self-adhesive gelled grounding pad was placed at the shaved back of each animal (group RFA). The surgical field was properly prepared, and a midline laparotomy was performed under aseptic conditions to all animals. The left lateral hepatic lobe was exposed, and the tip of the RFA electrode was inserted into liver parenchyma either to perform a RFA session (group RFA) or without any RF energy delivery (group Sham).Finally, the abdominal wall was closed in layers using 2-0 polyglactin suture.

### 2.4. Radiofrequency Ablation

A Radionics Cool-tip RFA System (Valleylab/Tyco Healthcare, Gosport, United Kingdom) consisting of a radiofrequency generator, a peristaltic perfusion pump, a grounding pad, and a single-shaft, 15 cm long needle electrode with a 2 cm exposure tip was used. The tip of the electrode was inserted into the hepatic parenchyma from the caudal surface of the lobe at a 90° angle. Sterile gauzes soaked in cold normal saline were placed around the lobe to prevent heat transmission to the surrounding tissues. The power delivered was 15 W for a 2 min period. The final tissue temperature reached between 50 and 60°C. During RFA sessions, the tip of the electrode was cooled by continuous perfusion of ice cold distilled water delivered by the peristaltic perfusion pump. Finally, the abdominal wall was closed in layers using 2-0 polyglactin suture.

### 2.5. Immunohistochemistry

Tissue specimens excised from the terminal ileum were fixed in formalin and embedded in paraffin according to standard histological procedures. Four-micron sections of representative blocks from each case were deparaffinized, rehydrated, treated with 0.3% H_2_O_2_ at room temperature for 15 min in methanol to block bioactivity of endogenous peroxidase, and immunostained employing the UltraVision HRP/DAB detection system (TP-125-HL, Thermo Scientific Inc., Germany) according to the manufacturer's instructions. Slides were then incubated for 60 minutes with the mouse monoclonal antibody CD68^+^ (AbD Serotec, UK), the mouse monoclonal antibody CD4^+^ (Thermo Scientific Inc., Germany), the mouse monoclonal antibody CD8^+^ (Thermo Scientific Inc., Germany), the rabbit polyclonal antibody MAdCAM-1 (Biorbyt, UK), the rabbit polyclonal antibody IL-6 (Santa Cruz Biotechnology Inc., USA), the mouse monoclonal antibody TNF*α* (Acris Antibodies Inc., USA), or the mouse monoclonal antibody NF*κ*Β (Santa Cruz Biotechnology Inc., USA), or at 1 : 100, 1 : 150, 1 : 150, 1 : 250, 1 : 500, 1 : 500, or 1 : 500 dilutions, respectively. Control slides were incubated for the same period with nonimmunized rabbit serum (negative control). Positive controls were also set up during the process. Bound antibody complexes were stained for 10 min with freshly prepared 0.05% diaminobenzidine (DAB). Sections were then briefly counterstained with Mayer's haematoxylin, mounted, and examined under a Nikon Eclipse 50i microscope. Antibody expression was graded in terms of the proportion of positively stained cells after scanning the entire section of each specimen according to the following scoring system: absence (0) for <10%; low (1) for 10–30%; moderate (2) for 31–70%; and high (3) for >70% positively stained cells. The score was the average of scores obtained by two independent operators.

### 2.6. Statistical Analysis

Before the beginning of the study, a sample size calculation was performed with 80% power and an error set at 0.01 (two sided). We estimated that a maximum of 6 rats per group would be required to detect a difference of 0.4 in CD4^+^ T-lymphocyte and 0.3 in NF*κ*B tissue expression scores with 0.2 and 0.2 standard deviations, respectively. Data were expressed as mean ± standard deviation. After normality of data was tested with the Kolmogorov–Smirnov test, those were subjected to analysis of variance (ANOVA). The Student *t*-test was used for comparisons between pairs of groups. A *p* < 0.05 was considered statistically significant.

## 3. Results

All animals survived the experimental period.

### 3.1. Immune Response

**Figure 1:**
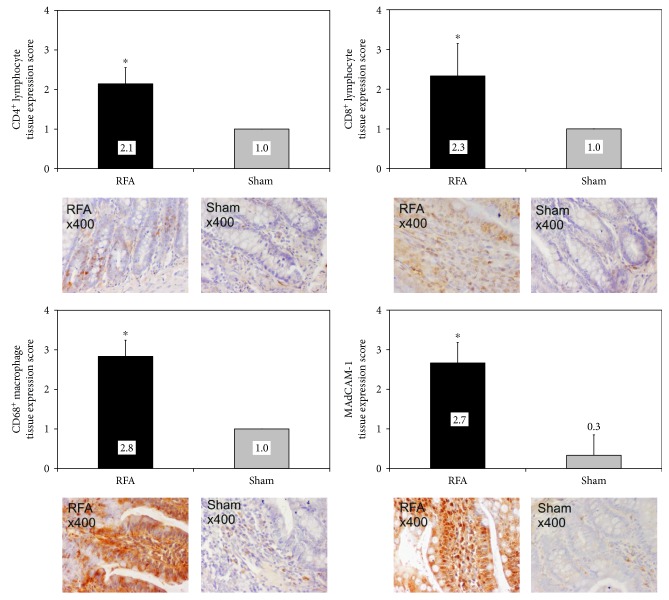
Ileal tissue immunohistochemical expression of CD4^+^ T-lymphocytes, CD8^+^ T-lymphocytes, CD68^+^ macrophages, and MAdCAM-1 in rats subjected to either liver RFA (group RFA) or sham operation (group Sham). ^∗^*p* < 0.001 versus group Sham.

Expression of immune response biomarkers in ileal tissue was mild in the sham-operated animals. On the other hand, biomarkers were overexpressed in the RFA group. In specific, expression was moderate for CD4^+^ and CD8^+^ T-lymphocytes, while high for CD68^+^ macrophages and MAdCAM-1. Tissue expression score was significantly higher (*p* < 0.001) in the group RFA compared to the group Sham for all biomarkers (Figure 1).

### 3.2. Inflammatory Response

**Figure 2:**
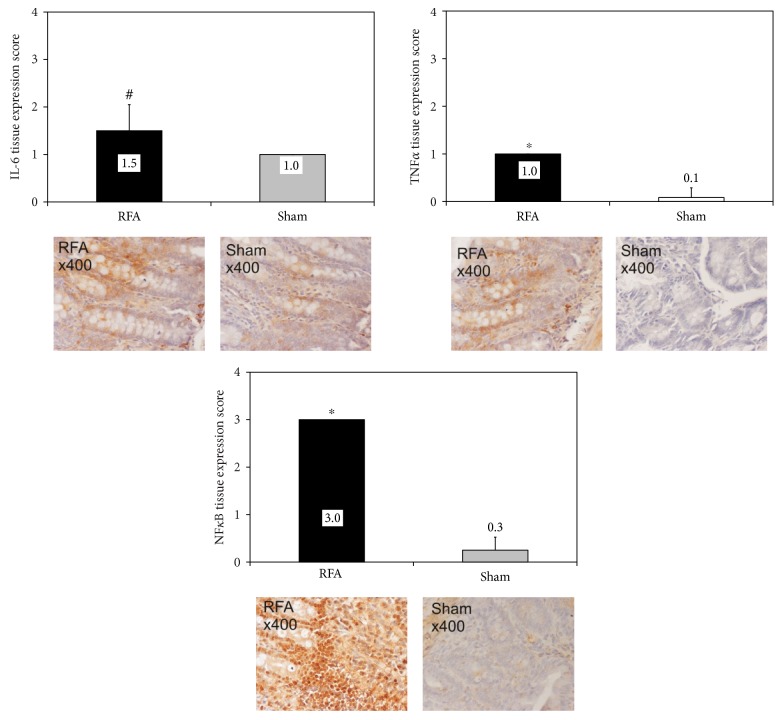
Ileal tissue immunohistochemical expression of IL-6, TNF*α*, and NF*κ*B in rats subjected to either liver RFA (group RFA) or sham operation (group Sham). ^#^*p* < 0.05, ^∗^*p* < 0.001 versus group Sham.

Expression of inflammatory response biomarkers was mild in the sham-operated animals. However, in the group RFA, expression was mild for TNF*α*, moderate for IL-6, while high for NF*κ*B. Tissue expression score was significantly higher in group RFA compared to group Sham (*p* < 0.05 for IL-6, *p* < 0.001 for TNF*α* and NF*κ*Β) for all biomarkers (Figure 2).

The immunohistochemical expression of all specific biomarkers had several localization patterns including nuclear, supranuclear, subnuclear, and mostly diffuse cytoplasmic.

## 4. Discussion

Disruption of gut barrier integrity and penetration of intraluminal microorganisms into the intestinal mucosa activate local immune and inflammatory response mechanisms beyond the steady-state condition. Extended liver RFA has been shown to lead to disruption of intestinal epithelium integrity with subsequent translocation of bacteria and endotoxins [13, 14] providing new insight to the pathogenetic mechanism responsible for postliver RFA infectious complications. According to the present experimental study, extended liver RFA exacerbated the immune and inflammatory responses of the gut mucosa, as documented by the upregulation of specific tissue biomarkers.

Intestinal epithelium cells regulate mucosal immune homeostasis by interacting with commensal bacteria. Contact of intraluminal microbes, with the intestinal epithelium, stimulates mucosal immune cells to produce proinflammatory cytokines such as TNF, lymphotoxin, and IL-6; these contribute to the formation of secondary lymphoid tissues and the homeostasis of mucosal immune systems, such as the production of IgA and the differentiation of T-cells. Interestingly, despite the constant biological signals of commensal bacteria to the intestinal tissue, activation of mucosal immune cells is low, maintaining a steady-state homeostasis known as “physiological inflammation.” Under conditions that lead to temporary disruption of the gut epithelial barrier, intraluminal bacteria invade the mucosa, resulting to recruitment and activation of proinflammatory mucosal immune cells for the initiation of acute inflammation [1]. Bacteria that manage to reach the lamina propria get phagocyted by intestinal macrophages. Intestinal macrophages are characterized by potent phagocytic and bactericidal activities but, unlike other tissue macrophages, do not secrete proinflammatory cytokines, preventing thus excessive inflammatory reaction [16]. Indeed, in the present study, there was a substantial upregulation of CD68^+^ macrophages in the intestinal mucosa of rats subjected to liver RFA with only low to moderate expression of inflammatory response markers TNF*α* and IL-6, respectively.

Intraepithelial lymphocytes take part in the local immunosurveillance of the intestinal epithelium. CD4^+^ and CD8^+^ T-lymphocytes located in nonmucosal lymph nodes get activated, migrate to the intestinal wall, and transiently accumulate in the intraepithelial compartment [17, 18]. Within the lamina propria, the majority of T-cells are CD4^+^, with a smaller population of CD8ab^+^ cells [19]. CD4^+^ lymphocytes, when activated, secrete cytokines, such as interferon-*γ* and TNF*α*, which increase transcellular intestinal permeability and paracellular intestinal permeability through a MLCK-dependent tight junction disruption or alternatively via dysregulation of occludin expression [20]. Receptors and their ligands necessary for T-cell homing in the intestine include MAdCAM-1, integrin a4b7, lymphocyte function-associated antigen-1, intercellular adhesion molecule-1, very late antigen-4 (a4b1), vascular cell adhesion protein 1, CCR9, CCL25, P-selectin glycoprotein ligand-1, and P-selectin [19]. In the present study, CD4^+^ and CD8^+^ T-lymphocytes, as well as MAdCAM-1, were upregulated in the intestinal mucosa 48 hours post-RFA.

NF*κ*B signaling cascade plays an important role in intestinal epithelium homeostasis. Accelerated epithelial apoptosis is associated with the development of intestinal inflammation as a result of commensal bacteria invasion through epithelial barrier breaches, hyperactivation of mucosal immune cells, and subsequent exacerbation of inflammatory conditions [1]. While intrinsic NF*κ*B signaling negatively regulates apoptosis of intestinal epithelial cells, excessive NF*κ*B activation promotes detrimental intestinal inflammation [21]. In the present study, activated NF*κ*B was markedly expressed in the intestinal mucosa. Under this perspective, *Ν*F*κ*B upregulation could further clarify the mechanism of increased crypt cell apoptosis noted after extended liver RFA in previous experimental studies [13, 22].

Bile plays an important role in maintaining gut mucosa immunologic homeostasis. According to animal studies, obstructive jaundice downregulates the numbers of CD4^+^ and CD8^+^ T-lymphocytes and MAdCAM-1 expression in the lamina propria [23]. Although, extended liver RFA has been shown to cause a reduction in bile flow rate, the findings of the present study did not reveal any suppression of gut immune response [15].

Production of proinflammatory cytokines by the inflamed intestine gives new insight to the mechanism responsible for the development of systemic inflammatory response syndrome (SIRS) and multiorgan injury secondary to liver RFA [22]. So far, translocation of enteral bacteria to distant organs via the lymph or the systemic circulation secondary to gut barrier failure has been considered the dominant pathogenetic mechanism leading to SIRS and multiple organ dysfunction syndrome (MODS) in a variety of systemic stress conditions, such as burn injury and severe pancreatitis [8, 24]. Extended liver RFA has been shown to lead to an elevation of proinflammatory cytokine levels in the systemic circulation and to tissue damage of adjacent and distant to the liver organs [22]. In addition, enteral bacteria were detected in proximal and remote organs confirming the involvement of translocating bacteria in the phenomenon [13]. Recent data provide evidence for the diffusion of nonmicrobial, proinflammatory, tissue injurious agents from the gut, through the lymph, to remote organs as an alternative pathway leading to SIRS and MODS [25]. The cytokine producing intestine of animals subjected to liver RFA could support this hypothesis, highlighting the key role of the inflamed gut in the development of SIRS and multiorgan injury secondary to liver RFA. Gut ischemia is considered the primary link through which splanchnic hypoperfusion is transduced from a hemodynamic into an immunoinflammatory event leading to the release of biologically active factors into the mesenteric lymphatics [13]. Hemodymanic studies have revealed a reduction in superior mesenteric artery blood flow rate at the early postliver RFA period [25] suggesting that gut ischemia-reperfusion is a key phenomenon in the cascade of events leading to oxidative stress-related apoptosis of intestinal epithelium cells [13, 14] and probably to the exacerbated immunoinflammatory response of the gut noted in the present study.

In conclusion, extended liver RFA evoked substantial immune, while moderate inflammatory response of the intestinal mucosa. This effect could be the result of epithelial gut barrier disruption and/or gut ischemia-reperfusion injury, while contributing to further disruption of the intestinal epithelium barrier. It addition, it highlights the potential role of the inflamed intestine to the mechanism responsible for the development of SIRS and multiorgan injury after extended liver RFA. These findings further clarify the effect of liver RFA on gut barrier function and could provide useful information in designing preventive and therapeutic strategies to increase the safety of the procedure.
